# Supplementary material for: β-Carotene from the Alga Dunaliella bardawil Decreases Gene Expression of Adipose Tissue Macrophage Recruitment Markers and Plasma Lipid Concentrations in Mice Fed a High-Fat Diet
Source: Mar Drugs. 2022 Jun 29;20(7):433. doi: 10.3390/md20070433 (PMC9316718; doi:10.3390/md20070433)
Supplement: Supplementary file 1 [file marinedrugs-20-00433-s001.zip › marinedrugs-1692870-supplementary.pdf]

Supplementary material

Β-Carotene from the Alga *Dunaliella Bardawil* Decreases Gene Expression of Adipose Tissue Macrophage Recruitment Markers and Plasma Lipid Concentrations in Mice Fed a High-Fat Diet

Nir Melnikov [†,1,2], Yehuda Kamari [1,2], Michal Kandel-Kfir [1], Iris Barshack [1,2], Ami Ben-Amotz [3], Dror Harats [1,2], Aviv Shaish [1,4,†] and Ayelet Harari [1,†,*]

[1] The Bert W. Strassburger Metabolic Center, Sheba Medical Center, Tel-Hashomer 5262000, Israel; nir.melnikov@sheba.gov.il (N.M.); yehuda.kamari@sheba.gov.il (Y.K.); michal.kandel.kfir@sheba.gov.il (M.K.-K.); iris.barshack@sheba.gov.il (I.B.); dror.harats@sheba.gov.il (D.H.); aviv.shaish@sheba.gov.il (A.S.)
[2] The Sackler Faculty of Medicine, Tel-Aviv University, Tel-Aviv 6997801, Israel
[3] N.B.T., Nature Beta Technologies LTD, Eilat 8851100, Israel; amiba@bezequin.net
[4] The Department of Life Sciences, Achva Academic College, Shikmim 7980400, Israel
* Correspondence: ayelet.harari@sheba.health.gov.il; Tel.: +972-3-5302006
† These authors contributed equally to this work.

Table of contents:

Table S1. D06040702 Research Diets high-fat rodent diet formulation.

Table S2. Real-Time PCR primers and probes (mouse).

Figure S1. Three-week-old male mice were fed an HFD (n = 15) or an HFD-BC (n = 13) for 23 weeks. NMR analyzed body composition.

Figure S2. IPGTT (A) after 16 wk (4h fast, n = 7). Blood glucose (B) and plasma insulin concentrations (C) after 18 wk (4h fast, n = 8). Values are mean ± SEM. IPGTT, Intraperitoneal glucose tolerance test.

Figure S3. eWAT adipocyte area (A) and liver NALFD activity score (B-E) after 23 weeks (n = 4-5). Values are median and quartiles (A) or mean ± SEM (B-E).

Figure S4. Representative images of eWAT (A) and liver (B) sections stained with H&E. All images were acquired under 20x magnification.

Table S1. D06040702 Research Diets high-fat rodent diet formulation.

	g	kcal%
Protein	26.2	20
Carbohydrate	25.0	20
Fat	34.9	60
Total		100
kcal/g	5.24	
**Ingredient**	**g**	**kcal**
Casein, 30 Mesh	200	800
L-Cystine	3	12
Corn Starch	0	0
Maltodextrin 10	125	500
Sucrose	68.8	275
Cellulose, BW200	50	0
Soybean Oil	25	225
Lard	245	2205
Mineral Mix S10026[1]	10	0
DiCalcium Phosphate	13	0
Calcium Carbonate	5.5	0
Potassium Citrate, 1 H_2O	16.5	0
Vitamin Mix V13001 (No added Vitamin A)[1]	10	40
Choline Bitartrate	2	0
FD&C Yellow Dye #5	0.025	0
FD&C Red Dye #40	0	0
FD&C Blue Dye #1	0.025	0
**Total**	**773.85**	**4057**

[1] Compositions of Mineral Mix S10026 and Vitamin Mix V10001 were previously described by Takemura et al. [1]. Vitamin Mix V13001 and Vitamin Mix V10001 are identical, except that Vitamin Mix V13001 does not contain added vitamin A.

1. Takemura N, Hagio M, Ishizuka S, Ito H, Morita T, Sonoyama K. Inulin prolongs survival of intragastrically administered Lactobacillus plantarum no. 14 in the gut of mice fed a high-fat diet. J Nutr. 2010;140(11):1963–9.

Table S2. Real-Time PCR primers and probes (mouse).

Gene	Universal ProbeLibrary #	Forward primer (5'-3')	Reverse primer (5'-3')
Gapdh	29 (4687612001)	ttcaccaccatggagaagg	cacacccatcacaaacatgg
Tnfα	49 (4688104001)	tcttctcattcctgcttgtgg	ggtctgggccatagaactga
Il-6	78 (4689011001)	tctaattcatatcttcaaccaagagg	tggtccttagccactccttc
Il-1β	78 (4689011001)	tgtaatgaaagacggcacacc	tcttctttgggtattgcttgg
Mcp-1	69 (4688686001)	aactctcactgaagccagctct	gtggggcgttaactgcat
Cd68	78 (4689011001)	ttctgctgtggaaatgcaag	tcacggttgcaagagaaaca
Ucp1	34 (4687671001)	tcaggattggcctctacgac	ttaagccggctgagatcttg
Pgc1α	34 (4687671001)	tgaaagggccaaacagagag	gtaaatcacacggcgctctt

Gene	PrimeTime qPCR Probe Assay	Forward primer	Reverse primer
Gapdh	Mm.PT.39a.1	aatggtgaaggtcggtgtg	gtggagtcatactggaacatgtag
Pparγ	Mm.PT.58.31161924	ctgctccacactatgaagacat	tgcaggttctactttgatcgc

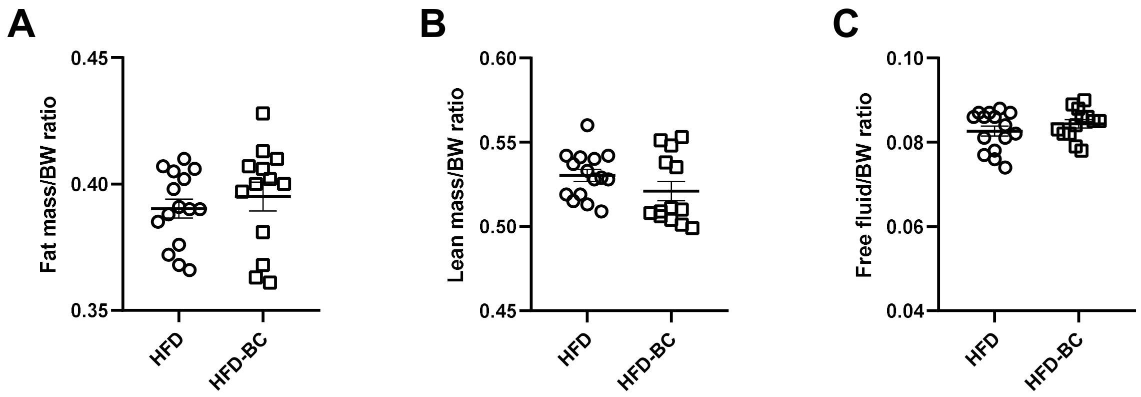

Figure S1. Three-week-old male mice were fed an HFD (n = 15) or an HFD-BC (n = 13) for 23 weeks. Body composition (A - fat mass, B - lean mass, C - free fluid) was analyzed by NMR following 14 weeks of treatment. The NMR instrument was calibrated according to the manufacturer's instructions and the mice were weighed and inserted into the test chamber (minispec Live Mice Analyzer (LF50), Bruker Optics, Inc.). Values are means ± SEM. BW, body weight; HFD, high-fat diet; HFD-BC, high-fat diet supplemented with *Dunaliella bardawil*.

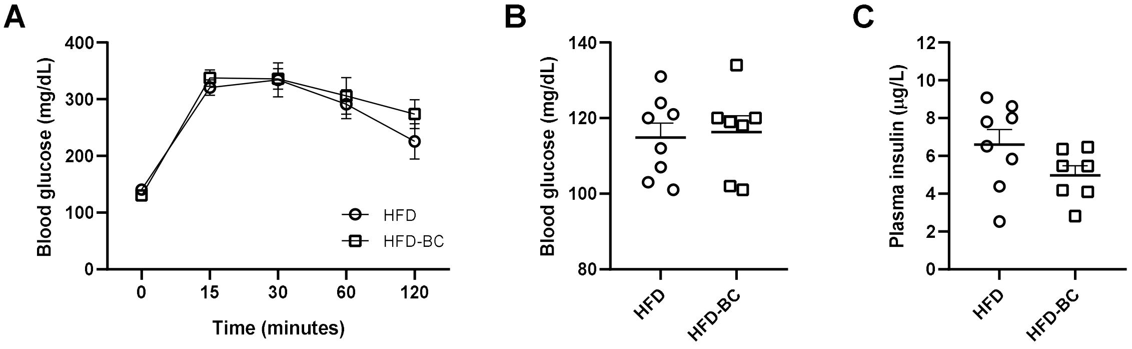

Figure S2. IPGTT (A) after 16 wk (4h fast, n = 7). Blood glucose (B) and plasma insulin concentrations (C) after 18 wk (4h fast, n = 8). Values are mean ± SEM. IPGTT, Intraperitoneal glucose tolerance test.

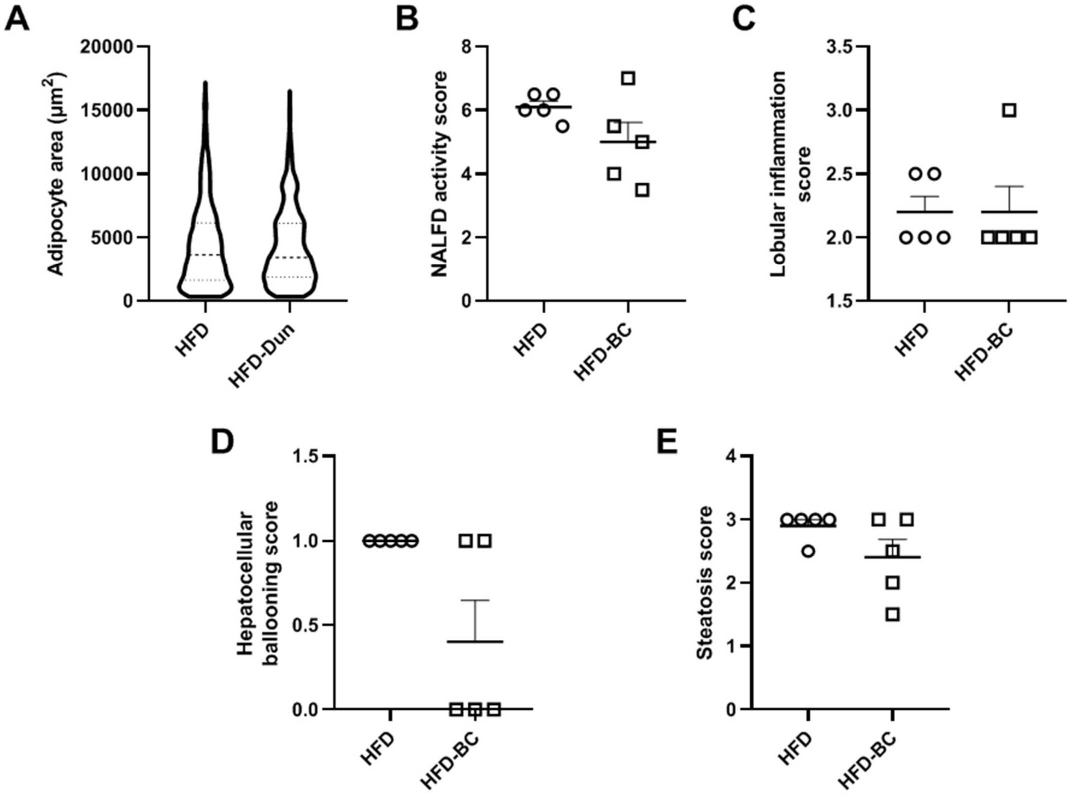

Figure S3. eWAT adipocyte area (A) and liver NALFD activity score (B-E) after 23 weeks (n = 4-5). Values are median and quartiles (A) or mean ± SEM (B-E).

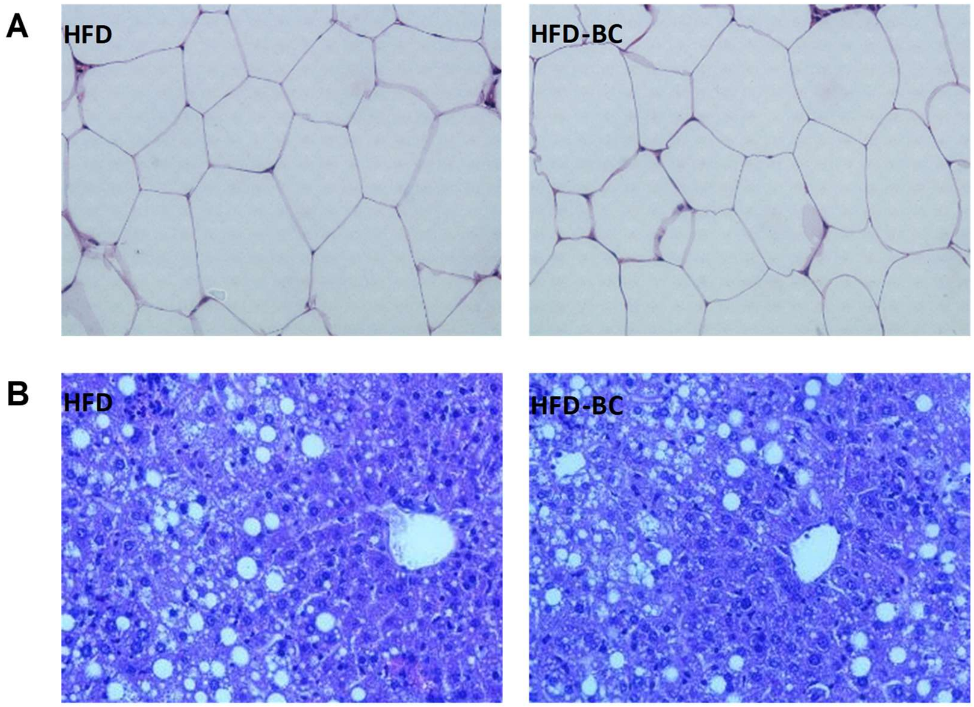

Figure S4. Representative images of eWAT (A) and liver (B) sections stained with H&E. All images were acquired under 20x magnification.
